# Immunization with a Recombinant Protein of *Trichinella britovi* 14-3-3 Triggers an Immune Response but No Protection in Mice

**DOI:** 10.3390/vaccines8030515

**Published:** 2020-09-09

**Authors:** Anna Stachyra, Sylwia Grzelak, Katarzyna Basałaj, Anna Zawistowska-Deniziak, Justyna Bień-Kalinowska

**Affiliations:** Witold Stefański Institute of Parasitology, Polish Academy of Sciences, 00-818 Warsaw, Poland; sylwia.grzelak@twarda.pan.pl (S.G.); kbasalaj@twarda.pan.pl (K.B.); anna.zawistowska@twarda.pan.pl (A.Z.-D.); jkalinowska@twarda.pan.pl (J.B.-K.)

**Keywords:** *Trichinella britovi*, 14-3-3 protein, immune response, cytokines, immunization

## Abstract

14-3-3 proteins are present in all eukaryotic organisms and are ubiquitously expressed in a broad range of tissues and cellular compartments. They are regulatory adapter proteins that play key roles in a variety of signaling pathways, and have been proposed as suitable targets for the control and detection of certain parasites. *Trichinella britovi* is a widely-distributed parasitic nematode, transmitted through ingestion of meat products containing invasive larvae. The present study describes the cloning and expression of Tb14-3-3, and investigates the immunological and protective potential of the recombinant protein. Immunization of mice with rTb14-3-3 triggered an IgG response, and significant differences, in the profiles of secreted cytokines observed in vitro, between experimental groups. Nonetheless, neither specific antibodies, nor increased secretion of IFNγ, IL-4, and IL-10 cytokines, conferred greater protection against infection. No reduction in larval burden was observed during recovery at 48 dpi. Additionally, rTb14-3-3 was not recognized by sera from the infected control mice, except for one, suggesting some mismatch between native and recombinant Tb14-3-3 antigenic sites. Therefore, before 14-3-3 can be considered a potential tool for *Trichinella* detection and vaccination, more research regarding its target proteins, and actual specific function, is needed.

## 1. Introduction

*Trichinella* is a genus of parasitic nematodes infecting mainly mammals, but it is also known to affect reptiles and predatory birds [1,2,3]. As infection mainly occurs through the ingestion of meat containing muscle larvae (ML), its natural hosts are carnivores and omnivores, however occasional infections are also observed in herbivores, such as horses [4,5,6]. The entire life cycle of *Trichinella* is completed in a single host: the molting and maturing of larvae in the intestine, followed by the mating of the adult worms (Ad), and the subsequent release of newborn larvae (NBL), which migrate to the muscle tissues [7]. It is important to emphasize that *Trichinella* has a unique life cycle among parasitic nematodes, and is therefore a very interesting research object. The parasite does not demonstrate any free-living stages and is not secreted to the environment. It acts as an intestinal parasite during the early stages of infection, similar to other families of parasitic nematodes. At the later phase, the invasive larvae migrate to the host’s muscles, where they form nurse cells and wait to be ingested by the next potential host, which is a very unique life strategy [8]. This uniqueness was recently confirmed at the genomic level, in a broad comparative study, where the *Trichinella* genus, placed in a small clade I, was found to be very distinct from other nematodes and helminths [9]. It leads to the conclusion that *Trichinella* species have developed some specific mechanisms at the cellular and molecular level, which enable them in successful completion of the life cycle.

Human trichinellosis is still one of the most important parasitic zoonoses, and is commonly caused by consumption of undercooked meat products containing invasive larvae [7,10,11]. Although human infections have recently fallen in number due to high biosecurity standards and strict veterinary inspections, the *Trichinella* population continues to be maintained among wildlife, and continual risk of transmission exists, to both domestic animals and humans. As the predominant species of *Trichinella* in the European area are *T. spiralis* and *T. britovi*, further research concerning *T. britovi* is clearly needed due to problematic diagnosis and treatment of trichinellosis. Serological tests often give high rates of false negative results during the early stage of infection, when the parasite is in the intestinal stage, and is most susceptible to anthelmintic treatment. Specific adaptive responses to infection are mostly developed during the parenteral phase. Encapsulated larvae are far less susceptible to anthelmintics, and may persist in the host tissues for a long time [12]. Although the search for a *Trichinella* vaccine has continued for several years, no effective formulation has yet been developed.

14-3-3 proteins are acidic regulatory adapter proteins that are present in all eukaryotic organisms, including yeast, protozoa, animals, and plants. There are several identified isoforms, traditionally assigned as β, ε, η, γ, θ, ζ, σ, based on early research in mammals. Isoforms are present in different tissues, and perform a number of common and specific functions. Being highly conserved, 14-3-3s play important roles in numerous cellular processes [13,14]. They regulate enzyme activity and the subcellular localization of target proteins, and play key roles in a variety of signaling pathways, including metabolism, cell division, stress responses, protein trafficking, and immune responses [15,16,17]. They can modulate functions of target proteins by changing their localization, and suppressing or promoting their association with other proteins. They demonstrate three major modes of action: enabling conformational changes of the target proteins, masking target proteins interaction sites (and thus providing e.g., protection against enzymes), and anchoring target proteins close to each other [18,19,20].

The 14-3-3 polypeptide forms nine antiparallel α-helices. The amino acid sequence contains alternate conserved and variable regions. Invariant residues are mostly found in the internal pocket, while differentiating residues are placed at the surface. These are specific to the isoforms and affect their interaction with target proteins. Variable regions also usually correspond to the unstructured ends of the helices [17,21,22]. To be fully active, 14-3-3 proteins form C-shaped homo- and hetero-dimers, possessing a large negatively-charged pocket capable of binding a large number of phosphorylated-serine target proteins. Several hundred have already been identified. However, phosphorylation-independent binding modes have also been confirmed among 14-3-3 proteins [13,18,23,24].

In the field of parasitology, 14-3-3 proteins have been assumed to play an important role in parasite proliferation and survival [25,26]. The earliest identification of a 14-3-3 protein isoform in a parasitic organism was in *Schistosoma mansoni* [27]. So far, the most investigated 14-3-3 proteins have been those found in some trematodes [28,29,30,31,32,33] and apicomplexa protozoa [34,35,36,37,38]. Subsequent studies have described 14-3-3 proteins present in the cestodes [39,40,41,42] and nematodes [43,44,45,46]. The 14-3-3s are well conserved among both parasitic and free living nematodes [43]. Phylogenetical analysis indicates that they can be classified as zeta-like isoforms and, interestingly, they share more similarity with certain 14-3-3 zeta-like isoforms, from arthropods, plants, or mammals, than with 14-3-3s from other helminths, such as flukes or tapeworms. Even less similarity can be found with 14-3-3s of protozoan parasites (Figure S1). Usually a single 14-3-3 isoform can be found in protozoa, and these demonstrate the closest similarity to mammalian epsilon isoform. However, they are larger proteins and contain additional N-terminus sequences, with different structures [22,34,35,36,37,38]. In tapeworms and flukes, three or four isoforms of 14-3-3 can be found, these being classified as epsilon-like, zeta-like, beta-like, and gamma-like [26,42,47,48].

The different isoforms of 14-3-3 proteins could demonstrate varying specific functions between distinct groups of parasites, especially in terms of their role in the host–parasite interface. The *Schistosoma, Echinococcus, Haemonchus,* and *Toxoplasma* 14-3-3 proteins have been detected as components of excretory–secretory antigen, suggesting their direct role in host–parasite interactions and the induction of the key immune response. However, immunization studies based on the 14-3-3 of nematode *Haemonchus contortus* indicated little protective effect, and were not very promising [44]. In contrast, those based on the 14-3-3 of *Echinococcus multicularis* or *Schistosoma* demonstrated a significant level of protection in mouse models [29,32,41]. The protective potential of 14-3-3 against platyhelminths has not been extensively evaluated in large animal models [39], and existing studies have found it to be rather ineffective, despite early optimistic findings [30]. Only one study has been conducted with *Trichinella* 14-3-3 protein; immunization with recombinant *T. spiralis* 14-3-3 yielded promising results in mice, with a 46% reduction of larval burden observed after challenge [49].

A recent analysis the of somatic protein extract of *T. britovi* ML, based on 2-D immunoblotting and liquid chromatography-tandem mass spectrometry (LS-MS/MS), performed by our group [50], revealed a spot that reacted with the sera of *T. britovi* infected pigs, and was identified as 14-3-3 protein zeta. Its molecular mass was approximately 25 kDa, which corresponded with the expected mass of the protein. This result was also in general agreement with those of the studies given above, indicating that 14-3-3 is a promising immunoreactive protein, worth subsequent research. Therefore the present study describes its expression in a verified eukaryotic system, i.e., *Pichia pastoris*, known to be a suitable host for the production of *T. britovi* protein [51], and evaluates its immunological potential against *T. britovi* infection in a mouse model.

## 2. Materials and Methods

### 2.1. Ethics Statements

The study was approved by the First Local Ethical Committee for Scientific Experiments on Animals in Warsaw, Poland (resolution no.: 020/2016, 23 March 2016). All efforts were made to minimize suffering, and all applicable international, national, and/or institutional guidelines for the care and use of animals were followed. In order to collect material from infected and/or immunized mice, animals were humanely euthanized by cervical dislocation.

### 2.2. T. britovi Parasite and Mouse Model

The reference strain of *T. britovi* (ISS002) had been maintained by several passages in male C3H mice at the Institute of Parasitology, PAS. The muscle larvae used for challenge infection of mice and as a source of genetic material for cloning were recovered from the previously-infected mice by HCl-pepsin digestion [52]. The animals were housed in a temperature-controlled environment at 24 °C with 12-h day-night cycles, and received food and water ad libitum.

### 2.3. Sequence Analysis

Amino acid sequences were aligned using CLUSTALW. The hypothetical structure was simulated by RaptorX server (http://raptorx.uchicago.edu/) using human 14-3-3ζ (PDB ID 4N84 and 5WXN) as templates. Prediction of N-glycosylation sites was performed using NetNGlyc v1.0 server (http://www.cbs.dtu.dk/services/NetNGlyc/) and GlycoPred server (https://comp.chem.nottingham.ac.uk/glyco/).

### 2.4. Cloning of Recombinant 14-3-3 Protein

*T. britovi* ML were obtained for total RNA isolation with a Total RNA mini Plus kit (A&A Biotechnology, Gdynia, Poland). The RNA template was then used for cDNA synthesis with a Maxima First Strand cDNA Synthesis Kit for RT-qPCR (Thermo Scientific, Waltham, MA, USA), according to the manufacturer’s protocol. The DNA coding for *T. britovi* 14-3-3 was amplified by PCR from the ML cDNA with 14-3-3 specific primers (Forward: 5′-AGGCATCGATAATGACCGAAAAGGAAGAC-3′, Reverse: 5′-TATCTAGAACCTGCCCAGCGGCTGTAT-3′). Primers were designed according to the nucleotide sequence of *T. spiralis* 14-3-3 zeta protein (GenBank no. XP003378934). Tb14-3-3 coding DNA was sequenced and subcloned into the yeast expression vector pPICZαC, with the His-tag at C-terminus (Thermo Scientific, Waltham, MA, USA). The correct reading frame of the recombinant plasmid was confirmed by DNA sequencing, using the vector flanking primers, 5’AOX1 and 3’AOX1.

### 2.5. Expression and Purification of Recombinant 14-3-3 Protein in P. pastoris

*Pichia pastoris* cells (X33 strain) were transformed with recombinant plasmids by electroporation. X33 transformant selection was performed using medium containing Zeocin, and integration of the 14-3-3 gene into the *P. pastoris* genome was confirmed by PCR. The recombinant Tb14-3-3, with the C-terminus his-tag, was expressed under induction with 0.5% methanol for 72 h in 200 mL of buffered methanol-complex medium (BMMY), and then purified by immobilized metal ion affinity chromatography using Protino Ni-NTA agarose (Macherey-Nagel, Duren, Germany). Protein samples were analyzed by sodium dodecyl sulfate–polyacrylamide gel electrophoresis (SDS PAGE) and Western blotting; the concentration was measured using a Pierce™ BCA Protein Assay Kit (Thermo Scientific, Waltham, MA, USA).

### 2.6. SDS-PAGE and Western Blot Analysis

The proteins were separated in 12% BisTris polyacrylamide gels. After electrophoresis, the gels were either stained with Coomasie brilliant blue or the proteins were transferred to a nitrocellulose membrane (Bio-Rad, Hercules, CA, USA) for Western blotting. The recombinant protein was detected using monoclonal Anti-polyHistidine—Peroxidase antibody (diluted 1:4000; Sigma, St. Louis, MO, USA). The blots were viewed with Super Signal Western Pico Chemiluminescent Substrate (Thermo Scientific, Waltham, MA, USA). The recombinant and native protein was also detected using sera from immunized or infected mice (diluted 1:400 or 1:100) and a secondary anti-mouse IgG antibody conjugated with HRP (diluted 1:8000; Abcam, Cambridge, UK). Additionally, native PAGE electrophoresis was performed in 12% BisTris polyacrylamid gel without SDS and Tris-Glycine buffer without SDS, and stained with Coomasie brilliant blue.

### 2.7. Glycoproteins Staining and Enzymatic Deglycosylation

To determine whether rTb14-3-3 was glycosylated in the *P. pastoris* cells, a Pierce Glycoprotein Staining Kit (Thermo Scientific, Waltham, MA, USA) was used. The protein samples transferred to the nitrocellulose membrane were stained according to the manufacturer’s protocol. Soybean trypsin inhibitor and horseradish peroxidase were used as non-glycosylated and glycosylated controls, respectively. Additionally, recombinant protein was digested with Endo H (New England Biolabs, Ipswich, MA, USA) in denaturing conditions, as described in the manufacturer’s instructions, and visualized by SDS-PAGE.

### 2.8. Immunization and Challenge Infection

Eight-week old male C3H mice were divided into three groups: one 14-3-3 immunized group of 12 animals, and two control groups of six animals each. The 14-3-3 group was immunized subcutaneously with 25 μg of rTb14-3-3, emulsified with the Alhydrogel adjuvant (InvivoGen, San Diego, CA, USA) in a total volume of 100 μL (antigen/Alhydrogel = 75/25 *v/v*). The mice were boosted with the same dose after one week. The control groups were injected with PBS or Alhydrogel using the same regimen. One week after the final vaccination, half of the animals from each group (i.e., six or three) were sacrificed, and the blood and spleens were harvested for immunological tests. The remaining mice from each group were challenged with 500 *T. britovi* ML in a total volume of 200 µL, administered orally. Seven weeks (48 days) after infection, all infected mice were sacrificed, blood and spleens were harvested, and *T. britovi* muscle larvae were recovered by HCl-pepsin digestion. The level of protective immunity was calculated based on the reduction in the number of recovered larvae, per gram (LPG) of muscles, obtained from all mice immunized with rTb14-3-3, compared with control group values. Sera from all mice blood samples were isolated and frozen at −20 °C for further analysis.

### 2.9. Determination of Serum Specific Antibodies

The levels of Tb14-3-3-specific antibodies (IgG as well as subclasses IgG1 and IgG2a) in the mouse serum samples were measured by indirect ELISA one week after final immunization, and seven weeks after challenge infection, using rTb14-3-3 as a coating antigen. Briefly, ELISA plates were coated with 4 μg/mL of rTb14-3-3 protein and incubated at 4 °C overnight. Next, diluted mouse serum samples (1:100) were added and incubated at 37 °C for one hour. The plates were then incubated with HRP-conjugated antibody goat anti-mouse IgG, IgG1, or IgG2a (1:80,000 or 1:60,000; Abcam, Cambridge, UK), for detection of 14-3-3-specific antibodies. An enzymatic color reaction was generated using TMB substrate (3,3′,5,5′-Tetramethylbenzidine; Sigma, St. Louis, MO, USA), and the absorbance value was measured at 450 nm using a Synergy HT microplate reader (BioTek, Hercules, CA, USA). The cut-off value of ELISA was evaluated on the basis of the mean optical density (OD) value plus three standard deviations (SD) of negative group serum samples.

### 2.10. Cytokine Analysis

To measure the specific cellular response, the spleens were harvested from vaccinated animals one week after final immunization, and seven weeks after challenge infection. The spleens obtained from the mice of the immunized group were pooled from pairs of randomly-selected mice, the splenocytes were disassociated using a 70 μm cell strainer and then suspended in complete RMPI medium (Biowest, Riverside, MO, USA). The spleens obtained from the mice of the control groups were pooled together from groups of three mice and subjected to the same procedure. In order to lyse the erythrocytes, the splenocytes were incubated in 5 mL RBC lysis buffer (Thermo Scientific, Waltham, MA, USA) for 10 min. The cell suspension was centrifuged at 250× *g* at room temperature for seven minutes. The cell pellets were washed in RPMI medium and then resuspended in complete RPMI medium containing 10% FBS and penicillin/streptomycin (Biowest, Riverside, MO, USA), and counted.

For the cytokine stimulation assay, the splenocytes were seeded in a 24-well culture plate (Corning, Corning, NY, USA) at 5 × 10^6^ cells per well in 1000 μL medium. The cells were then stimulated with 15 μg/mL rTb14-3-3 and incubated for 72 h at 37 °C in a humidified atmosphere of 5% CO_2_. Cells stimulated with 5 ug/mL Concavalin A were included as positive controls. Non-stimulated cells were included as negative controls. After 72 h, the cells were pelleted by centrifugation at 1000× *g* for 10 min and the supernatants were collected for cytokine measurement. Supernatant samples were tested for levels of IL-2, IL-4, IL-10, IFN-γ, using a Mouse Th1/Th2 uncoated ELISA kit (Thermo Scientific, Waltham, MA, USA).

### 2.11. Statistical Analysis

Statistical analysis was performed using Statistica 6 software (StatSoft, Tulsa, OK, USA). Data were expressed as means ± standard deviation (SD). Differences among groups were analyzed by one-way analysis of variance (ANOVA). A value of *p* < 0.05 was considered significant.

## 3. Results

### 3.1. Cloning, Expression, and Purification of Recombinant Tb14-3-3 Protein

**Figure 1:**
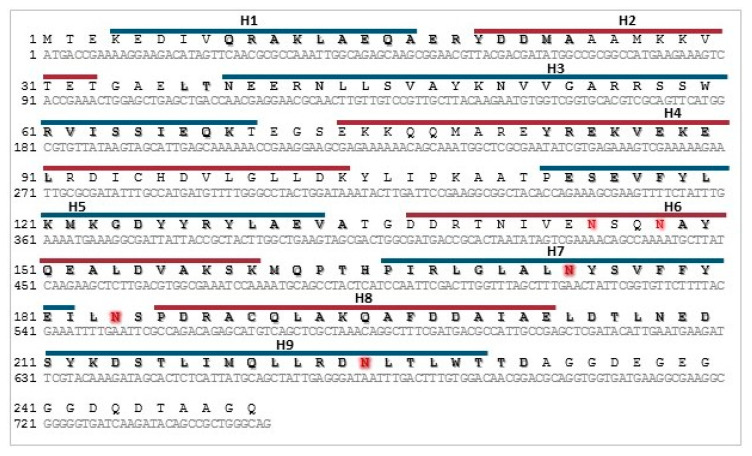
Schematic representation of cloned Tb14-3-3. Nucleotide and amino acid sequences are presented. Conserved regions of the protein chain are bolded (according to McGowan et al. [22]). Nine α-helices are marked with upper lines and numbered H1-H9. Potential N-glycosylation sites are highlighted in red.

As the amino acid sequences of the 14-3-3 proteins in the *Trichinella* genus share almost 100% identity, *T. spiralis* 14-3-3 zeta was used as the template for the design of the PCR primers. The DNA coding for Tb14-3-3 (GenBank no. KRY57650) was successfully amplified and subcloned into the expression vector. The sequenced gene revealed one amino acid substitution (K116E), between the hypothetical and cloned Tb14-3-3. An analysis of the amino acid sequence using NetNGlyc v1.0 server determined two potential N-glycosylation sites, these being in the conserved regions of α-helix 7 and α-helix 9. Analysis using GlycoPred server determined five potential N-Glycosylation sites: in addition to the two above, it also identified two sites in the variable region of α-helix 6, and one site in the conserved coil between α-helix 7 and α-helix 8. It also identified 15 potential O-glycosylation sites. The cloned Tb14-3-3 is presented in Figure 1.

Expression of the 14-3-3 gene in X33 *P. pastoris* strain was induced with the addition of 0.5% methanol every 24 h until the final time of induction, i.e., 72 h. As the level of expression was high, no subsequent optimization was performed. The recombinant His-tagged protein was purified using affinity chromatography under native conditions.

### 3.2. Characterization of rTb14-3-3 Protein

**Figure 2:**
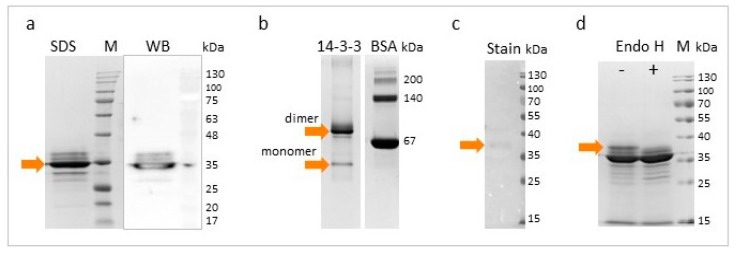
Analysis of rTb14-3-3 protein produced in *P. pastoris* culture. (**a**) Purified recombinant protein (7 µg/lane) visualized with SDS-PAGE and Anti-His Western blot. M-marker (**b**) Native PAGE of rTb14-3-3 (6 µg/lane) and bovine serum albumin (BSA), used as a molecular standard (molecular weight of BSA monomer and dimer is indicated). The predicted rTb14-3-3 monomer and dimer forms are marked with arrows. (**c**) Colorimetric detection of glycoproteins. Protein samples (6 µg/lane) were separated on SDS-PAGE, and transferred to the nitrocellulose membrane. Slight coloration, corresponding to the top band of rTb14-3-3, is marked with an arrow. (**d**) SDS-PAGE after deglycosylation of rTb14-3-3 with Endo H. Protein samples (8 µg/lane) were incubated with Endo H enzyme (+), or without Endo H (−), in denaturing conditions. The top band, which was no longer present after the enzymatic digestion, is marked with an arrow.

The theoretical molecular weight of the His-tagged Tb14-3-3 was predicted as 31.1 kDa. SDS-PAGE analysis found rTb14-3-3 to display an intense band of approximately 35 kDa. Moreover, some less visible additional upper and lower bands were observed. These were suspected to represent different forms, glycoisoforms, and possible degradation products (Figure 2a). Subsequent visualization with native PAGE revealed that the majority of the protein was present as a homodimer form (ca. 70 kDa), and a low amount of monomer form was also visible (Figure 2b).

To examine the N-glycosylation of recombinant Tb14-3-3, glycoprotein staining was performed followed by Endo H digestion. The results indicate that only a small portion of the protein pool was glycosylated in *Pichia* cells, most likely with high mannose oligosaccharides. Enzymatic digestion did not result in a great deal of change of the SDS-PAGE electropherogram; only the top band, which was slightly stained during the glycoprotein staining procedure, was no longer present (Figure 2c,d).

### 3.3. Humoral Antibody Response Induced by Immunization of Mice with rTb14-3-3 Formulated with Alhydrogel

**Figure 3:**
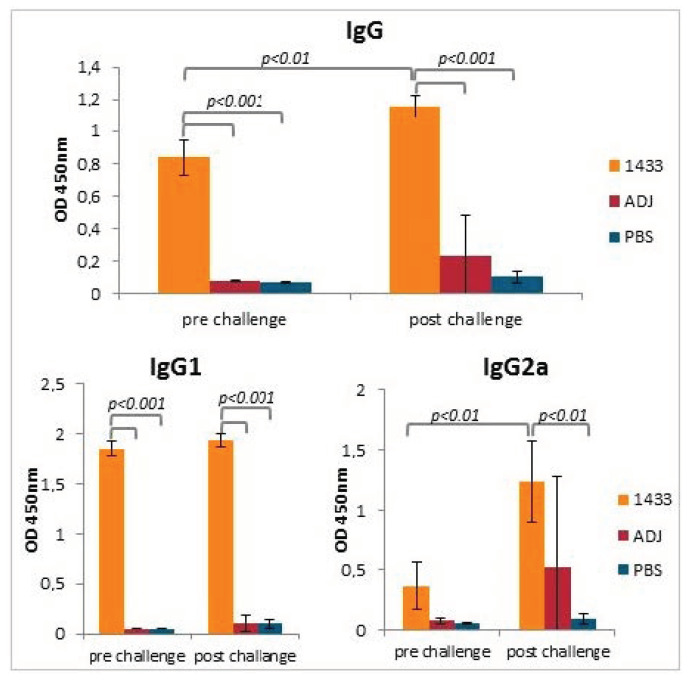
Antibody response in mice after immunization and challenge infection. Anti-Tb14-3-3 total IgG level, and IgG1 and IgG2a subclass levels, in the sera of immunized mice, measured by ELISA, are shown on graphs. Experimental groups: 1433-group injected with rTb14-3-3, ADJ-group injected with adjuvant only, PBS-group injected with PBS. Significantly different groups are marked with brackets and *p* value is indicated. Bars represent mean values from six individuals (*n* = 6) from the immunized group, or three individuals *(n* = 3) from control groups ±SD.

The purified rTb14-3-3 was tested for its immunogenicity in a mouse model. The humoral responses in experimental animals were evaluated by ELISA, using recombinant Tb14-3-3 as an antigen. The total IgG level analysis indicated that all mice from the immunized group responded well to the vaccine, while no antibodies recognizing 14-3-3 were detected in control groups. After the challenge infection, the level of anti-14-3-3 total IgG in the immunized group was significantly higher than before infection; however, no anti-14-3-3 antibodies were detected in the control groups (Figure 3). When the IgG1 and IgG2a subclasses were tested, a relatively high level of IgG1 antibodies and a low level of IgG2a antibodies were observed in the immunized group, after final immunization. After infection with *T. britovi*, the level of IgG1 remained similar, but the level of IgG2a was significantly higher than before infection. Interestingly, a high level of IgG2a was also detected in one individual from the adjuvant control group (Figure 3).

**Figure 4:**
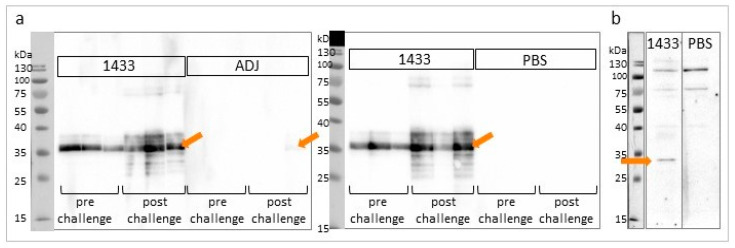
Immunoblot analysis of sera from experimental mice. (**a**) Recombinant Tb14-3-3 (3.8 μg/lane) detection using mouse sera and secondary anti-mouse IgG antibodies. Experimental groups, named as previously described, and serum collection time (pre or post challenge) are indicated. Specific signals for the 35 kDa band are marked with arrows. (**b**) Native Tb14-3-3 detection using experimental mice sera. *T. britovi* muscle larvae (ML) somatic protein extract (10 µg/lane) was incubated with serum from immunized (1433) and control (PBS) mice and secondary anti-mouse IgG antibodies. The specific signal for the 30 kDa band is marked with an arrow.

Western blot total IgG analysis confirmed the presence of seroconversion in all mice from the immunized group, while no antibodies recognizing 14-3-3 protein were detected in the control groups. After the challenge infection, the anti-14-3-3 total IgG signal in the immunized group was stronger, indicating a specific boost of the response. Interestingly, the signal was also visible in one individual from the adjuvant control group, confirming the earlier ELISA result (Figure 4a).

The mice sera obtained after immunization were also subjected to Western blot for the detection of native 14-3-3 in *T. britovi* somatic protein extract. The presence of a band at approximately 30 kDa was assumed to be a specific signal of 14-3-3 (Figure 4b).

### 3.4. Cytokine Profiles of Stimulated Splenocytes from Mice Immunized with rTb14-3-3

**Figure 5:**
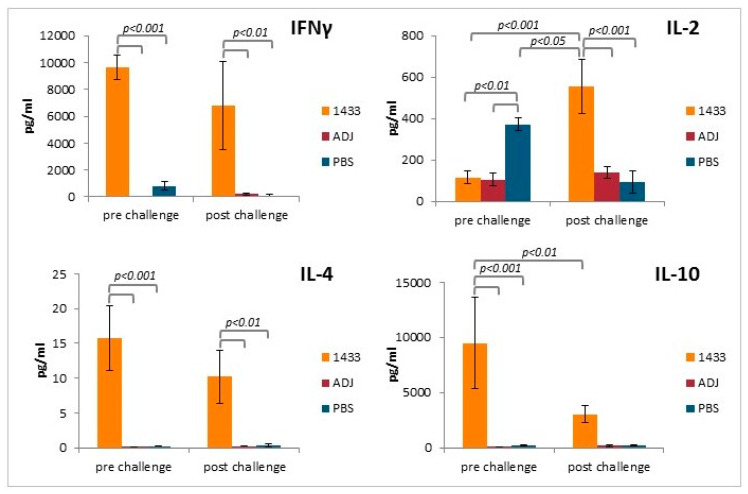
Cytokine profiles of mouse splenocytes after immunization and challenge. Supernatants harvested after 72 h of incubation were analyzed for detection of secreted cytokines IFNγ, IL-2, IL-4, and IL-10, using a Mouse Th1/Th2 uncoated ELISA kit. Experimental groups are indicated as previously described. Significantly different groups are marked with brackets and *p* value is indicated. Bars represent means ±SD from three splenocyte cultures, each prepared from two pooled spleens from mice from the immunized group (*n* = 3), or means ±SD from three repeats of splenocyte cultures, prepared from three pooled spleens from mice from the control group (*n* = 3).

To further investigate whether a Th1/Th2 mixed response was induced following Tb14-3-3 vaccination, the levels of selected cytokines were measured in the supernatants of the stimulated splenocyte cultures. IFNγ was found to be present at high levels in the supernatants of splenocyte cultures from mice immunized with rTb14-3-3, collected before and after challenge, but at low levels in those of the control groups (Figure 5). In contrast, IL-2 was present at low levels in the supernatants of stimulated splenocyte cultures from mice immunized with Tb14-3-3, but at significantly higher levels after challenge. In addition, a high level of IL-2 was detected in the supernatants of stimulated splenocyte cultures from uninfected PBS control mice. In the remaining groups, the level of IL-2 was low (Figure 5). IL-4 was present at high levels in the supernatants of splenocyte cultures from the immunized mice, before and after challenge, but was only present at the limit of detection in the supernatants of the control groups (Figure 5). IL-10 was also found to be present at high levels in the supernatants of splenocyte cultures from the immunized mice, but these were significantly lower after the challenge infection, and were not different from the control groups; again at the limit of detection (Figure 5). In general, rTb14-3-3 was a poor stimulant for splenocytes from non-immunized mice (infected or not), but results confirm that immunization with recombinant protein induced a mixed Th1/Th2 response.

### 3.5. Protective Immunity Induced by rTb14-3-3 Protein

Seven weeks (48 days) after infection, all mice were sacrificed and *T. britovi* muscle larvae were recovered from individual mice and counted. The larval burden was compared between all groups: no reduction of muscle larvae was observed in the immunized group and no significant differences were observed between groups (Table 1).

## 4. Discussion

14-3-3 proteins are ubiquitous in eukaryotes and play many essential roles in cellular processes. The present study describes an immunological analysis of *T. britovi* 14-3-3 protein, encoded by cloned ML cDNA. Recombinant Tb14-3-3 was produced in a yeast system and then used for the immunization of C3H mice; a *Trichinella* model used in our lab. PAGE analysis indicated that the rTb14-3-3 protein most likely forms a functional dimer, and is almost free of attached glycans, despite having up to five potential N-glycosylation sites. One week after final immunization, a high level of antibodies was detected in mice sera, including both IgG1 and IgG2a, with IgG1 being relatively dominant. Secretion of selected cytokines, IFNγ, IL-4, and IL-10, was detected in the stimulated splenocytes of the immunized animals, confirming induction of Th1/Th2 immunological responses.

However, immunization with the Tb14-3-3 protein did not bring any visible protective effect against infection, measured as a reduction in ML, in the selected experimental model and schedule. Furthermore, IgG analysis showed that the antibodies induced in the control groups during infection did not recognize the recombinant Tb14-3-3 protein, and the reasons for this are unclear. It is possible that the antigenicity of native Tb14-3-3 is relatively weak, or that it could not be exposed for a long time. Furthermore, the available epitopes of 14-3-3, and their recognition by immune cells, could be influenced by the binding of different target client proteins from the parasite or the host. In contrast, the native 14-3-3 present in the protein extract was recognized by antibodies induced after immunization, suggesting that the recombinant protein demonstrates stronger antigenicity and broader availability of its epitopes.

The recombinant 14-3-3 protein from *T. spiralis* was previously investigated as a potential diagnostics and vaccine candidate by Yang et al. [45,49]. The protein was first detected by 2-D immunoblot as a highly immunodominant antigen, reacting with early infection sera of pigs and mice. Similar results have been obtained in immunoproteomic analysis of *T. britovi* [50]. Immunological activity between recombinant Ts14-3-3 and sera from infected animals has previously been confirmed by Western blot examination [45]. However, our present ELISA and Western blot findings do not indicate any such reaction between rTb14-3-3 and the sera of infected mice in control groups, apart from in one individual. Yang et al. [45] also report that native *T. spiralis* antigen reacts in Western blot with serum of immunized mice. A similar reaction was also detected in the present study, suggesting that some native and recombinant epitopes are common, and the antibodies induced after immunization with recombinant protein have a higher ability to recognize native antigen than the inverse configuration. This phenomenon is in agreement with the observation of a specific antibody boost, especially IgG2a, after infection of animals previously immunized with r14-3-3.

In an immunization study by Yang et al. [49], both Th1 and Th2 immune responses were stimulated, resulting in a significant reduction in ML after challenge with *T. spiralis*. Unfortunately, IgG and cytokine analysis was performed only after immunization. No data concerning the effect of immunization on the host response was acquired after infection. However, unlike Yang et al., our present findings suggest that induction of both Th1 and Th2 immune responses failed to visibly affect parasite development, and ML numbers were not reduced.

The differences in outcomes between the two studies could be attributed to the differences in the expression system, i.e., bacteria vs. yeasts, which could affect the epitopes, conformation, and activity of the antigen. It is important to note that the 14-3-3 family proteins may play many roles, depending on the specific situation and conditions. The exact function of Tb14-3-3, which includes interactions with proper target proteins, could require highly-specific posttranslational modifications such as phosphorylation, acetylation, glycation, or glycosylation [17,34]. These modifications may not be correct in the heterologous-expression host. Our analysis identified some level of glycosylation, but only a small part of the protein pool had been glycosylated, and it is unclear whether this accurately reflects the glycan content of the native protein pool during infection. Further studies are needed to determine the occupation of potential sites, and to characterize the oligosaccharide chains present.

The differences could be also caused by the immunization schedule, which was established based on our experience in the field, and more significant effects could be possibly achieved using a higher dosage, or different adjuvants, time spaces, or dose numbers. Yang et al. [49] immunized mice with a total number of three doses, while our group immunized mice with a total number of two doses. However, in our opinion, it is unlikely that 14-3-3 protein could be a highly immunogenic antigen, since it is a well-conserved protein, and the structural differences between particular isoforms (*Trichinella* and mammalian isoform zeta) are very subtle. It is not possible that a strong antibody response would occur against parasite 14-3-3, because it would affect the activity of the host 14-3-3 zeta protein. In addition, the actual function of Tb14-3-3, also could require the presence of other *Trichinella* proteins, which are produced during infection. In this case, immunization with a single recombinant protein, without its accompanying elements, would not induce an adequate immunological response against the parasite. This assumption seems to be supported by our data, where antibodies induced after *T. britovi* infection were generally not able to recognize the recombinant antigen, and no effects of stimulation were observed in splenocytes from the infected animals. On the other hand, native 14-3-3 has previously been recognized as an immunoreactive protein in proteomical analysis [50], and was also detected by the serum of experimentally vaccinated mice.

## 5. Conclusions

It can be assumed that Tb14-3-3 protein has some important function in parasitic invasion, since it is immunoreactive and can induce immunological responses in the host. However, no current knowledge exists about the actual specific functions of *Trichinella* 14-3-3 proteins. In addition, no direct comparison can be made with other parasites, since their 14-3-3s are usually classified as other isoforms, and their specific roles in parasitic invasion and life cycle can likely differ. Until we better understand the true function of *Trichinella* 14-3-3 during infection, the target client proteins, and the significance in the stages of the parasite life cycle, 14-3-3 will remain an unlikely candidate as a potential vaccine or diagnostic antigen. Therefore, further studies are needed to evaluate the targets and modes of action of Tb14-3-3.

## Figures and Tables

**Table 1:** *T. britovi* muscle larvae (ML) recovered from infected mice by HCl-pepsin digestion.

Group	Muscle Larval Burden (LPG)
14-3-3 ^a^	12,117.7 ±1656.6
ADJ ^b^	14,739 ±1984.9
PBS ^b^	11,649 ±2395.9

The results are means from six or three individuals with ±SDs indicated; ^a^ *n* = 6, ^b^ *n* = 3.

## Supplementary Materials

The following are available online at https://www.mdpi.com/2076-393X/8/3/515/s1, Figure S1: Sequence of *T. britovi* 14-3-3 aligned with selected 14-3-3 protein isoforms.
