# Supplementary material for: Immunization with a Recombinant Protein of Trichinella britovi 14-3-3 Triggers an Immune Response but No Protection in Mice
Source: Vaccines (Basel). 2020 Sep 9;8(3):515. doi: 10.3390/vaccines8030515 (PMC7564242; doi:10.3390/vaccines8030515)
Supplement: Supplementary file 1 [file vaccines-08-00515-s001.pdf]

```
TB --------------------------------------------------------------MTEKEDIVQRAKLAEQAERYDDMAAAMKKVTETGA--- 35
TS --------------------------------------------------------------MSEKEDIVQRAKLAEQAERYDDMAAAMKKVTETGA--- 35
HC -------------------------------------------------------------MADNKDELVQRAKLAEQAERYDDMAQSMKKVTELGA--- 36
DI -------------------------------------------------------------MAENKDELVQRAKLAEQAERYDDMAQSMKRVTELGA--- 36
CE -------------------------------------------------------------MSDGKEELVNRAKLAEQAERYDDMAASMKKVTELGA--- 36
BX -------------------------------------------------------------MSDGKDELVQRAKLAEQAERYDDMAQSMKKVTELGS--- 36
AM -------------------------------------------------------------MSVDKEELVQRAKLAEQAERYDDMAAAMKAVTETGV--- 36
HV --------------------------------------------------------------MTDKEEQVQRAKLAEQAERYDDMAAAMKKVTESGA--- 35
HS ---------------------------------------------------------------MDKNELVQKAKLAEQAERYDDMAACMKSVTEQGA--- 34
SB ----------------------------------------------------MTTSWVLQCKDLSNTDLVHIAKLAEQAERYDDMAAAMKRYTEASG--- 45
HM ------------------------------------------------MVDVSSTEWLKDLPLRDRESLVYTAKCLEQAERFDDMAICMKEVTNFDQ--- 49
EG ------------------------------------------------MAELLSTEWLKDLPLKDRDSYVNTAKCLEQAERFDDMAVCMKEVTRFDK--- 49
EM --------------------------------------------------MAAITSWITDSGCKDHASLVSIAKLAEQAERYEDMAVAMKTIAEMGN--- 47
TG MVSTDIAFSSAKKSTRRPLLVFHRPARDFFVFLRFPSFFSGHFPSFPLLLSCVPVSTMAEEIKNLRDEYVYKAKLAEQAERYDEMAEAMKNLVENCLDEQ 100
ET ---------------------------------------------------------MIEDIKTLREEHVYRAKLAEQAERYDEMAEAMKNLVENCLDQN 43
GI -----------------------------------------------------------MAEAFTREDYVFMAQLNENAERYDEMVETMRKISGMEG--- 38
SM -------------------------------------------------------------MDELRERNIVLAKLCEQAERYDEMVKAMIEIATNTET-- 37

TB ---------ELTNEERNLLSVAYKNVVGARRSSWRVISSIEQKTE----GSEKKQQMAREYREKVEKELRDICHDVLGLLDKYLIPKAATPESEVFYLKM 122
TS ---------ELTNEERNLLSVAYKNVVGARRSSWRVISSIEQKTE----GSEKKQQMARDYREKVEKELRDICHDVLGLLDRYLIPKATTPESKVFYLKM 122
HC ---------ELSNEERNLLSVAYKNVVGARRSSWRVISSIEQKTE----GSEKKQQMAKEYREKVEKELRDICQDVLNLLDKFLIPKAGNPESKVFYLKM 123
DI ---------ELSNEERNLLSVAYKNVVGARRSSWRVISSIEQKTE----GSEKKQQMAKEYREKVEKELRDICQDVLNLLDKYLIPKAGNPESKVFYLKM 123
CE ---------ELSNEERNLLSVAYKNVVGARRSSWRVISSIEQKTE----GSEKKQQMAKEYREKVEKELRDICQDVLNLLDKFLIPKAGAAESKVFYLKM 123
BX ---------ELSNEERNLLSVAYKNVVGARRSSWRVISSIEQKTE----GSEKKQQMAKEYREKVERELRDICHDVLDLLDKFLIPKAGNPESKVFYLKM 123
AM ---------ELSNEERNLLSVAYKNVVGARRSSWRVISSIEQKTE----GSERKQQMAKEYREKVEKELREICYDVLGLLDKYLIPKASNAESKVFYLKM 123
HV ---------ELSNEERNLLSVAYKNVVGARRSSWRVISSIEQKTE----GSERKQTMAREYREKIEKELREICQDVLSLLDKYLIPKASNPESKVFYLKM 122
HS ---------ELSNEERNLLSVAYKNVVGARRSSWRVVSSIEQKTE----GAEKKQQMAREYREKIETELRDICNDVLSLLEKFLIPNASQAESKVFYLKM 121
SB ---------NLGNEERNLLSVAYKNVVGARRSAWRVIHGSEMKAV----NDCTKKQIAEEYRIKMEKELNTICNQVLALLEDYLLPNASPDDSKVFFLKM 132
HM ---------ELNNEERNLLSVAFKNVVGSRRNSYRVLSSRLSRTT----DAE-KQNLTKEYLEILQKELNDICTDVLKLIDERLYPKCSNAEGKVFYKKM 135
EG ---------ELNNEERNLLSVAFKNVVGSRRNSYRVLSSRLARTQ----DPE-KQALTREYLDILQKELNAICSDVLQLIEERLYPSCSNAEGKVFYKKM 135
EM ---------ELNNEERNLLSVAYKNVVGARRSSWRIMSSIAKKQA----GTP-LADQTDIYLKKVEEELTKICNDVLALLSKNLITEKIGAEAKIFYYKM 133
TG QPK-----DELSVEERNLLSVAYKNAVGARRASWRIISSVEQKELSK--QHMQNKALAAEYRQKVEEELNKICHDILQLLTDKLIPKTSDSESKVFYYKM 193
ET NSPPGAKGDELTVEERNLLSVAYKNAVGARRASWRIISSVEQKEANR--NHMANKALAASYRQKVENELNKICQEILTLLTDKLLPRTTDSESRVFYFKM 141
GI ---------ELSDKERNLLSVAYKNVIGPRRAAWRIVSSIEAKEKGRQKPNAKRIEQIRVYRQKIEKELSDICNDILKLLQEQFVPRSTNADAKVFYYKM 129
SM ---------ELTVEERNLLSVAYKNVIGARRSSWRIINSKESQDE---AKGSDEIHITKRFRKEVEKELDEICTSILNLLDNCLLPKAVSDESKVFLNKM 125

TB KGDYYRYLAEVATGDDRTNIVENSQNAYQEALDVAKSKMQPTHPIRLGLALNYSVFFYEILNSPDRACQLAKQAFDDAIAELDTLNEDSYKDSTLIMQLL 222
TS KGDYYRYLAEVATGDDRNSIVENSQSAYQEALDVAKSKMQPTHPIRLGLALNFSVFFYEILNSPDRACQLAKQAFDDAIAELDTLNEDSYKDSTLIMQLL 222
HC KGDYYRYLAEVASGEDRSSVVDKSQQSYQEAFDIAKDKMQPTHPIRLGLALNFSVFYYEILNAPDKACQLAKQAFDDAIAELDTLNEDSYKDSTLIMQLL 223
DI KGDYYRYLAEVASGDDRNVVVEKSQQSYQEAFDIAKDKMQPTHPIRLGLALNFSVFYYEILNAPDKACQLAKQAFDDAIAELDTLNEDSYKDSTLIMQLL 223
CE KGDYYRYLAEVASGDDRNSVVEKSQQSYQEAFDIAKDKMQPTHPIRLGLALNFSVFFYEILNAPDKACQLAKQAFDDAIAELDTLNEDSYKDSTLIMQLL 223
BX KGDYYRYLAEVATGDDRNAVVEKSQHSYQEAFDIAKDKMQPTHPIRLGLALNFSVFYYEILNAPDKACQLAKQAFDDAIAELDTLNEDSYKDSTLIMQLL 223
AM KGDYYRYLAEVATGETRNAVVEDSQKAYQEAFDIAKSKMQPTHPIRLGLALNFSVFYYEIINSPDKACQLAKQAFDDAIAELDTLNEDSYKDSTLIMQLL 223
HV KGDYYRYLAEVATGDNRTAVIDDSQKAYSEAFDIAKTQMQPTHPIRLGLALNFSVFYYEILNSPERACHLAKQAFDDAIAELDSLNEDSYKDSTLIMQLL 222
HS KGDYYRYLAEVAAGDDKKGIVDQSQQAYQEAFEISKKEMQPTHPIRLGLALNFSVFYYEILNSPEKACSLAKTAFDEAIAELDTLSEESYKDSTLIMQLL 221
SB QGDYYRYLAEVATDDARTEVVQKSLDAYTKATTAAEN-LPTTHPIRLGLALNFSVFYYEIQNNSAKACDLAKCAFDSAIAELDQLQDDSYKDSTLIMQLL 231
HM LGDYYRYKAENAKGEEHKAVVEASRLAYEDATKIA-ESLSCTHPIRLGLALNYSVFYYEIMNSPDRACQLAKKAFDDAVSDVDNANEESYKDSTLIMQLL 234
EG MGDYYRYKAENAKGEDHKQVVEASLKAYEEATEIANEKLSCTHPIRLGLALNYSVFYYEIMNSPDRACQLAKKAFDDAVSDVDSANDDSYKDSTLIMQLL 235
EM MGDYYRYLAEVQEGEQNDKSTEAAEEAYQKATSLAEAELSVTHPIRLGLALNFSVFYYEIKNMPEKACSLAKAAFDAAITEVDSIKDETYKDSTLIMQLL 233
TG KGDYYRYISEFSGEEGKKQAADQAQESYQKATETAEAELPSTHPIRLGLALNYSVFFYEILNLPQQACEMAKRAFDDAITEFDNVSEDSYKDSTLIMQLL 293
ET KGDYYRYISEFSNEEGKKASAEQAEESYKRATDTAEAELPSTHPIRLGLALNYSVFYYEILNQPQKACEMAKLAFDDAITEFDSVSEDSYKDSTLIMQLL 241
GI QGDYYRYLAEYSSGEDKEKIAGSALNAYNSAFEIS-QQLPPTHPIRLGLALNFSVFYYEILASPDRACELARKAFDAAITDLDKLTEESYKDSTLIMQLL 228
SM RGDYHRYRAEYSVGNQRKDAAENSLCAYKKAAEDAEK-LPVTHPIRLGLALNFSVFYYEILNNSEQACKCARVAFDSAIAELDTLSEESYKDSTIIMQLL 224

TB RDNLTLWTTDAGGDEGEGGGDQDTAAGQ-------- 250
TS RDNLTLWTTDNAGDEGDGGGEQDAPTGQ-------- 250
HC RDNLTLWTSDAAADDQDAGEQGEGAN---------- 249
DI RDNLTLWTSDTSADDQEGGEQAGEAGGN-------- 251
CE RDNLTLWTSDAATDDTDANETEGGN----------- 248
BX RDNLTLWTSDTAGEEQEGGNETGETGGN-------- 251
AM RDNLTLWTSDTQGDADEAQEGGDN------------ 247
HV RDNLTLWTSDATADDDQGQPDDNN------------ 246
HS RDNLTLWTSDTQGDEAEAGEGGEN------------ 245
SB RDNLTLWASDQTAEGDVENDG--------------- 252
HM RDNLALWTSDPEEIENNDGAE--------------- 255
EG RDNLALWTSDPEETENTEGAE--------------- 256
EM RDNLTLWNSECETDS--------------------- 248
TG RDNLTLWTSDLQADQQQQ----EGGEKPAEQADQ-- 323
ET RDNLTLWTSDLQTQEQQQQPVGEGAEAPKVEATEQQ 277
GI RDNLNLWVTDSAGDDNAEEK---------------- 248
SM RDNLTLWTSNSEGEKDTASPKGDKK----------- 249
```

Supplementary Figure 1. Sequence of *T. britovi* 14-3-3 aligned with selected 14-3-3 protein isoforms. 14-3-3 in nematodes (magenta) shows 88-96% conservation of sequence. 14-3-3 isoforms in Platyhelminthes (green) and protozoa (orange) share 63-67% and 54-66% similarity with Tb14-3-3, while human (blue), bee (yellow) and barley (brown) 14-3-3 zeta share 86%, 89% and 91% similarity with Tb14-3-3, respectively. TB - *Trichinella britovi* (clade I), TS - *Trichuris suis* (clade I) KFD65230.1, HC - *Haemonchus contortus* (clade V) CDJ94531.1, DI - *Dirofilaria immitis* (clade III) AHJ11155.1, CE - *Caenorhabditis elegans* (clade V) NP_509939.1, BX - *Bursaphelenchus xylophilus* (clade IV) ACZ13351.1, AM - *Apis mellifera* XP_006566159.1, HV - *Hordeum vulgare* BAJ95666.1, HS - *Homo sapiens* NP_001129171.1, SB - *Schistosoma bovis* (zeta) AAT39382.1, HM - *Hymenolepis microstoma* (zeta) CDS31680.1, EG - *Echinococcus granulosus* (zeta) XP_024353113.1, EM - *Echinococcus multicularis* AAM94864.1, TG - *Toxoplasma gondii* XP_002365409.1, ET - *Eimeria tenella* AAD02687.1, GI - *Giardia intestinalis* AAZ91664.1, SM - *Schistosoma mansoni* (epsilon) XP_018645303.1.
